# *E. coli* SbcCD and RecA Control Chromosomal Rearrangement Induced by an Interrupted Palindrome

**DOI:** 10.1016/j.molcel.2010.06.011

**Published:** 2010-07-09

**Authors:** Elise Darmon, John K. Eykelenboom, Frédéric Lincker, Lucy H. Jones, Martin White, Ewa Okely, John K. Blackwood, David R. Leach

**Affiliations:** 1 Institute of Cell Biology, University of Edinburgh, Kings Buildings, Edinburgh EH9 3JR, UK

**Keywords:** DNA, MICROBIO

## Abstract

Survival and genome stability are critical characteristics of healthy cells. DNA palindromes pose a threat to genome stability and have been shown to participate in a reaction leading to the formation of inverted chromosome duplications centered around themselves. There is considerable interest in the mechanism of this rearrangement given its likely contribution to genome instability in cancer cells. This study shows that formation of large inverted chromosome duplications can be observed in the chromosome of *Escherichia coli*. They are formed at the site of a 246 bp interrupted DNA palindrome in the absence of the hairpin nuclease SbcCD and the recombination protein RecA. The genetic requirements for this spontaneous rearrangement are consistent with a pathway involving DNA degradation and hairpin formation, as opposed to a cruciform cleavage pathway. Accordingly, the formation of palindrome-dependent hairpin intermediates can be induced by an adjacent DNA double-stand break.

## Highlights

► Palindrome-stimulated inverted chromosome duplication occurs in *E. coli* chromosome ► SbcCD and RecA protect the cell against inverted chromosome duplication ► DNA degradation by RecBCD is required for spontaneous inverted chromosome duplication ► Hairpin formation is stimulated by a DNA DSB induced in the region of a palindrome

## Introduction

DNA palindromes are present at sites of genome instability. They are implicated in gene deletion, gene amplification, chromosome fracture, and chromosome translocation, aberrations characteristic of hereditary disease and rearrangements observed in cancer cells (Coté and Lewis, 2008; Lewis and Coté, 2006; Lobachev et al., 2007; Tanaka et al., 2005; Tanaka and Yao, 2009). DNA palindromes are sequences with a rotational symmetry. A perfect palindrome is a DNA sequence that is immediately juxtaposed to an exact reverse complementary copy of itself, whereas an interrupted palindrome (also known as a closely spaced inverted repeat) contains a small unique sequence that separates the two inverted complementary copies (Lewis and Coté, 2006). Due to their symmetry, palindromes have the unique potential to form intrastrand base pairs in hairpin or cruciform DNA structures, and it is this potential that underlies their role in genomic instability (Lewis and Coté, 2006; Tanaka and Yao, 2009).

A common gross chromosomal rearrangement is a structure that is denoted here as an inverted chromosome duplication, in which part of a chromosome is lost and replaced by a duplication of the inverted remaining chromosome part. After its formation, this structure can be further rearranged and amplified by breakage-fusion-bridge cycles in organisms with linear chromosomes, centromeres, and nonhomologous end-joining (NHEJ) (Lobachev et al., 2007; McClintock, 1941; Tanaka and Yao, 2009). An important issue is the origin of the initial inverted chromosome duplication, and a widely accepted pathway is the joining of two sister chromatids by NHEJ at the site of a DNA break present prior to DNA replication (Kurahashi et al., 2006; Lewis and Coté, 2006; Lobachev et al., 2007). However, other studies have identified another pathway. A DNA palindrome inserted in a chromosome can initiate the formation of an inverted chromosome duplication, in which the palindrome itself is located at the center of the rearrangement (Butler et al., 1996; Haber and Debatisse, 2006; Tanaka and Yao, 2009). This finding suggests that naturally occurring palindromes can play important roles in the formation of these gross chromosomal rearrangements, and evidence exists that natural palindromes may be causative of rearrangements observed in human cancers (Tanaka and Yao, 2009).

**Figure 1:**
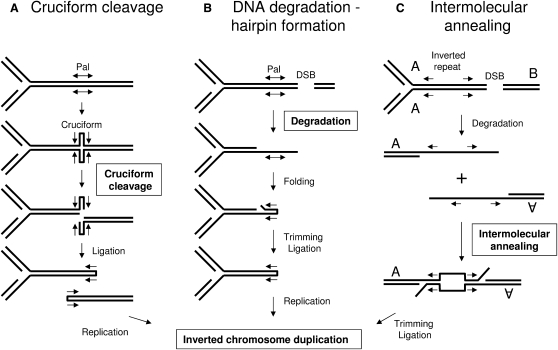
Main Hypotheses for the Role of DNA Palindromes and Inverted Repeats in the Formation of Inverted Chromosome Duplications (A) The palindrome (Pal) extrudes into a cruciform, which is cleaved across the four-way junction, resulting in two hairpin-capped ends. Replication of the hairpin-capped end results in an inverted chromosome duplication. (B) A DSB occurs near a palindrome on the origin-distal side. The free DNA ends are degraded by exonucleases. The single-stranded palindrome is able to fold back on itself, forming a hairpin-capped end. Replication of the hairpin-capped end results in an inverted chromosome duplication. When a DSB occurs on the origin proximal side, a hairpin-capped end also forms on the origin distal side of the palindrome but cannot be replicated into an inverted chromosome duplication. (C) A DSB occurs before replication of distant inverted repeats. The free DNA ends are degraded by exonucleases. Inverted chromosome duplications are formed by intermolecular single strand annealing (SSA) of the repeats.

Three main hypotheses have been proposed to explain the role of DNA palindromes and inverted repeats in the formation of inverted chromosome duplications (Figure 1). In the first mechanism (Figure 1A), the palindrome is proposed to extrude into a cruciform, which is cleaved across the four-way junction, leaving two hairpin-capped ends. Replication of the hairpin-capped ends forms inverted chromosome duplications. Cruciform extrusion in vivo has been inferred for perfect palindromes in plasmids (Coté and Lewis, 2008; Kurahashi et al., 2006; Lewis and Coté, 2006; Malagón and Aguilera, 1998; Sinden et al., 1991), in bacteriophage lambda (Davison and Leach, 1994), and in the mouse genome (Cunningham et al., 2003). Evidence consistent with this pathway has been obtained in a *Saccharomyces cerevisiae* plasmid, wherein formation of an inverted chromosome duplication involves the action of Mus81, a protein involved in the cleavage of four-way junctions (Coté and Lewis, 2008). Lobachev and collaborators have suggested that closely spaced Alu inverted repeats may also extrude into a cruciform in a *S. cerevisiae* chromosome and generate the same rearrangement (Lobachev et al., 2002; Narayanan et al., 2006).

In the second mechanism (Figure 1B), a DNA double-strand break (DSB) occurs near a palindrome, and the free DNA ends are degraded by exonucleases, which leaves the palindrome single stranded and able to fold back on itself, forming a hairpin-capped end (Butler et al., 1996; Tanaka and Yao, 2009). This can occur on either side of the palindrome, depending on the site of the DSB, to generate two hairpin-capped ends in a population of cells. Replication of the origin-proximal hairpin-capped end forms the inverted chromosome duplication. Here, perfect and interrupted palindromes are predicted to behave similarly, given that hairpin formation occurs in single-stranded DNA in which the spacer between inverted repeats is not expected to inhibit the reaction. This pathway is expected to be stimulated by DNA DSBs adjacent to the palindrome, which has been confirmed in several studies (Lin et al., 2001; Qin and Cohen, 2000; Tanaka et al., 2002; Tanaka et al., 2007). The first and second mechanisms are suppressed by the action of Mre11/Rad50/Sae2 (Coté and Lewis, 2008; Lobachev et al., 2002; Narayanan et al., 2006; Rattray et al., 2001; Rattray et al., 2005). In addition, the DSB-hairpin mechanism is expected to be opposed by homologous recombination via repair of the DSB. In *Schizosaccharomyces pombe*, the Rad51 recombination protein has been implicated in preventing the formation of an inverted chromosome duplication (Nakamura et al., 2008). In *S. cerevisiae*, RAD52 and Mre11 oppose the formation of long palindromes that are intermediates in a survival pathway for chromosomes with eroded telomeres in an ExoI- and telomerase-defective mutant (Maringele and Lydall, 2004).

Of interest, in *S. cerevisiae*, more distant inverted repeats can also induce the formation of inverted chromosome duplications by intermolecular single-strand annealing (SSA) of the repeats (Figure 1C) (Butler et al., 2002; Downing et al., 2008; VanHulle et al., 2007). Because this third pathway does not involve the formation of hairpins or cruciforms but does involve SSA, it is not opposed by Rad50/Mre11/Sae2 and is promoted by Rad52.

Whether all of these pathways exist remains to be clarified. The existence of two pathways, one that proceeds via an intramolecular reaction involving a hairpin or a cruciform intermediate (first and second mechanisms, characterized by an opposition by Rad50/Mre11/Sae2) and one that proceeds via intermolecular SSA (third mechanism, characterized by no opposition by Rad50/Mre11/Sae2), is strongly supported. However, the existence of both the cruciform and the hairpin pathways is still open to question.

In the chromosome of *Escherichia coli*, an interrupted 246 bp palindrome forms a hairpin on the lagging strand template at every replication cycle (Eykelenboom et al., 2008). The SbcCD complex (Rad50/Mre11 homolog) cleaves this hairpin, leaving a DSB. For the cell to survive, the DSB must be repaired by homologous recombination (Cromie et al., 2001; Dillingham and Kowalczykowski, 2008). First, the RecBCD complex unwinds and differentially degrades the two strands of the dsDNA ends until a specific DNA sequence, called *chi* (χ), is recognized. Following an encounter with the χ sequence, the 5′-3′ exonuclease activity of RecBCD is enhanced, and the 3′-5′ exonuclease activity is reduced, resulting in the formation of a 3′ overhanging end. Then, the ssDNA 3′ tail is coated by RecA (Rad51 homolog), which promotes strand invasion and strand exchange with a sister chromosome, creating a Holliday junction. Finally, RuvABC and RecG mediate branch migration and resolution of the Holliday junction to form two intact chromosomes.

The present work demonstrates that, in the absence of SbcCD and RecA proteins, a 246 bp or an 85 bp interrupted palindrome can induce the formation of an inverted chromosome duplication centered at the site of the palindrome. This gross chromosomal rearrangement was investigated by Southern blot analysis and fluorescence microscopy. The reaction is *recB* dependent, and hairpin formation is stimulated by an adjacent DSB, arguing that DNA degradation leaves a single-stranded palindromic sequence that forms a hairpin-capped end (Figure 1B). DNA replication of the hairpin structure produces the inverted chromosome duplication. Finally, this study shows that the events associated with the formation of inverted chromosome duplication can be visualized by fluorescence microscopy.

## Results

### Detection of Novel DNA Fragments Induced by a DNA Palindrome in the Absence of SbcCD and RecA

**Figure 2:**
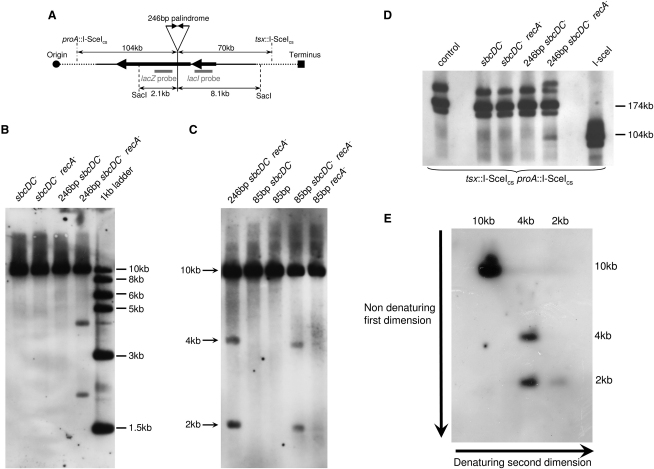
Study of Novel DNA Fragments in a *recA sbcDC* Mutant Containing a Palindrome Cells were grown in LB until an OD_600nm_ between 0.6 and 1. The DNA was digested by the SacI or I-SceI enzyme, and the region surrounding the palindrome insertion place in *lacZ* was studied by Southern blot analysis using an origin-proximal probe in *lacZ*. (A) Schematic representation of the region surrounding the palindrome insertion place in *lacZ*. The sizes indicated include the 246 bp interrupted palindromic sequence. (B) The strains used for the analysis were the *sbcDC* mutant (DL3325), the *sbcDC recA* mutant (DL3395), the *sbcDC* mutant containing the 246 bp palindrome (DL3326), and the *sbcDC recA* mutant containing the 246 bp palindrome (DL3396). The DNA was digested by the SacI enzyme. The 1kb ladder was used as a size standard. (C) The strains used for the analysis were the *sbcDC recA* mutant containing the 246 bp palindrome (DL3396), the *sbcDC* mutant containing the 85 bp palindrome (DL3835), the strain containing the 85 bp palindrome (DL3846), the *sbcDC recA* mutant containing the 85 bp palindrome (DL3856), and the *recA* mutant containing the 85 bp palindrome (DL3857). The DNA was digested by the SacI enzyme. (D) The strains used for the analysis contained two engineered I-SceI cutting sites (I-SceI_cs_) and were a control strain (DL2792), the *sbcDC* mutant (DL4461), the *sbcDC recA* mutant (DL4480), the *sbcDC* mutant containing the 246 bp palindrome (DL4466), the *sbcDC recA* mutant containing the 246 bp palindrome (DL4482), and a strain containing an additional I-SceI_cs_ in the *lacZ* gene (DL2849). The DNA was digested by the I-SceI enzyme and run on a PFGE. (E) DNA was digested by the SacI enzyme and studied by 2D native/denaturing electrophoresis before Southern blot analysis. The strain used for this analysis was the *sbcDC recA* mutant containing the 246 bp palindrome (DL3396).

The behavior of a 246 bp interrupted palindrome in the *lacZ* gene of the *E. coli* chromosome was investigated by Southern blot analysis. Chromosomal DNA of different *E. coli* strains was initially digested with the restriction enzyme SacI, which cleaves ∼2 kb from the palindrome on the origin-proximal side and 8 kb from the palindrome on the origin-distal side. DNA was analyzed using an origin-proximal probe in the *lacZ* gene (Figure 2A). Strikingly, in an *sbcDC recA* double mutant, the presence of the palindrome resulted in the appearance of two novel DNA fragments of ∼2 kb and 4 kb (Figure 2B). Two additional fragments of ∼104 kb and 208 kb also appeared when a larger part of the chromosome was studied using pulse-field gel electrophoreses (PFGE) (Figures 2A and 2D). Importantly, these DNA fragments were not detected in the DNA from an *sbcDC* single mutant carrying the palindrome, indicating that the absence of RecA is essential for their formation (Figures 2B and 2D). Because expression of SbcCD is lethal in a *recA* mutant strain containing the 246 bp interrupted palindrome (Eykelenboom et al., 2008), it was not possible to use this palindrome to test whether the formation of these novel fragments requires the absence of SbcCD in a *recA* mutant. However, the presence of an 85 bp interrupted palindrome does not impede the growth of a *recA* single mutant, indicating that this palindrome is not a target for SbcCD-mediated cleavage during replication. Therefore, an 85 bp interrupted palindrome was used to evaluate the role of SbcCD in the appearance of the novel fragments. Figure 2B shows that the levels of 2 kb and 4 kb fragments were dramatically decreased in the DNA from a *recA* single mutant carrying the 85 bp palindrome, whereas they were clearly visible in the DNA of an *sbcDC recA* double mutant. These results demonstrate that both RecA and SbcCD control the formation of the novel DNA fragments.

### The 2 kb Novel DNA Fragment Has a Hairpin-Capped End

Of note, the distances between the center of the 246 bp palindrome and the restriction enzyme sites on the origin-proximal side correspond to the sizes of the smaller novel DNA fragments on the Southern blots (2 kb for SacI and 104 kb for I-SceI). In the absence of the hairpin nuclease SbcCD, a hairpin might persist if formed by either mechanism A or B described in Figure 1. This hairpin-capped end would protect the DNA end from degradation by exonucleases and enable its detection by Southern blot analysis. To investigate whether the 2 kb fragment has a hairpin-capped end, DNA from an *sbcDC recA* double-mutant strain containing the 246 bp palindrome was studied on a two-dimensional (2D) neutral-denaturing gel (Figure 2E). Most of the fragment migrating at 2 kb under nondenaturing condition migrated as predicted for a 4 kb single-stranded DNA fragment following denaturation, indicating that the 2 kb fragment has a hairpin-capped end.

### Replication of the DNA Hairpin Leads to the Formation of an Inverted Chromosome Duplication

**Figure 3:**
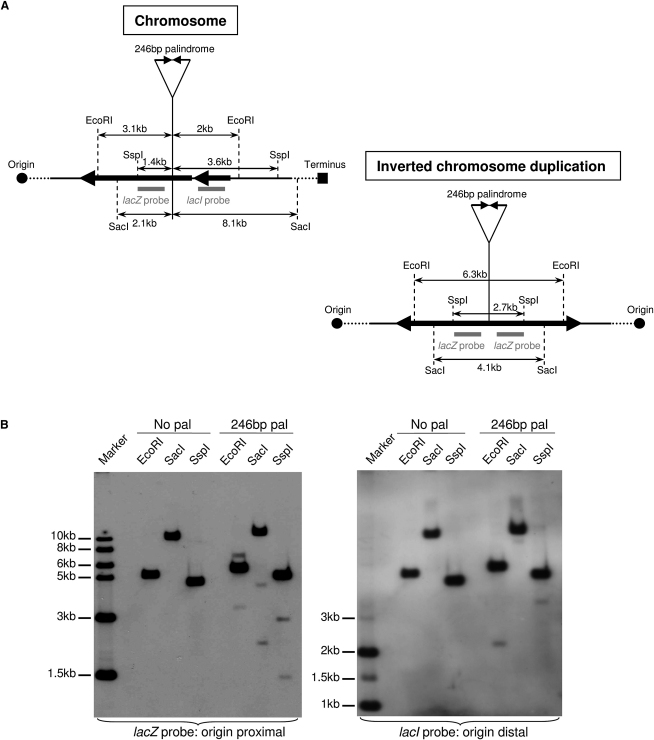
The Heaviest Novel DNA Fragment Is the Replication Product of the Hairpin (A) Schematic representation of the region surrounding the palindrome insertion place in *lacZ* before (left) and after (right) the formation of the inverted chromosome duplication. The sizes indicated include the 246 bp interrupted palindromic sequence. (B) Cells were grown in LB until an OD_600nm_ between 0.6 and 1. The DNA was digested by either EcoRI, SacI, or SspI enzyme, and the region surrounding the palindrome insertion place in *lacZ* was studied by Southern blot analysis using an origin-proximal probe in *lacZ* (left) or an origin-distal probe in *lacI* (right). The strains used for the analysis were the *sbcDC recA* mutant (DL3395) and the *sbcDC recA* mutant containing the 246 bp palindrome (DL3396). The 1 kb ladder was used as a size standard.

After DNA replication, the origin-proximal hairpin-capped end should become an inverted chromosome duplication, appearing on a Southern blot as a novel fragment double the size of the hairpin. In order to test whether the observed 4 kb novel fragment represents this inverted chromosome duplication, DNA from an *sbcDC recA* double-mutant strain containing the 246 bp palindrome was studied by Southern hybridization after digestion with SacI, SspI, or EcoRI, using the origin-proximal (*lacZ*) or an origin-distal (*lacI*) probe. A restriction map of the chromosomal *lac* region and a predicted restriction map of the inverted chromosome duplication are featured in Figure 3A. As shown in Figure 3B, the palindrome-dependent novel fragments displayed the sizes predicted for the hairpin-capped end and its replication product on the origin-proximal side. Of note, replication of the hairpin on the origin-distal side of the palindrome would require passage of the replication fork through the terminator region of the chromosome, so the origin-distal hairpin alone was observed when the DNA was probed using the origin-distal probe (*lacI*). Together, these data indicate that an *sbcDC recA* double-mutant strain containing the 246 bp palindrome allows the formation of an inverted chromosome duplication by replication of a hairpin generated at the site of the palindrome.

### Recombination Proteins Are Implicated in the Formation of the Chromosomal Rearrangements

**Figure 4:**
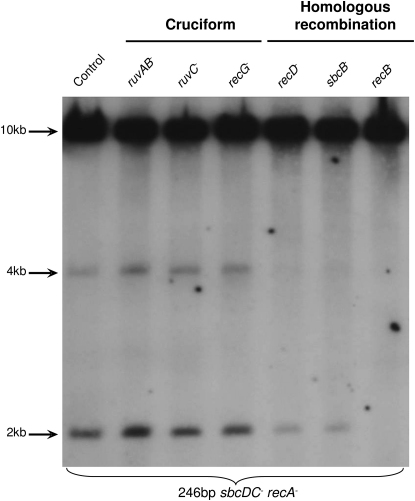
Recombination Proteins Are Involved in the Formation of the Novel DNA Fragments Cells were grown in LB until an OD_600nm_ between 0.6 and 1. The DNA was digested by the SacI enzyme, and the region surrounding the palindrome insertion place in *lacZ* was studied by Southern blot analysis using the origin-proximal probe in *lacZ*. The strains used for this analysis all contain the 246 bp palindrome and are *sbcDC recA* mutant (DL3396), *sbcDC ruvAB recA* mutant (DL3753), *sbcDC ruvC recA* mutant (DL3755), *sbcDC recG recA* mutant (DL3810), *sbcDC recD recA* mutant (DL3876), *sbcDC* s*bcB recA* mutant (DL3827), and *sbcDC recB recA* mutant (DL4136).

The detection of two hairpin-capped DNA molecules suggests that formation of the inverted chromosome duplication proceeds via a cruciform or hairpin intermediate, as expected in mechanisms A or B of Figure 1. In order to distinguish between these pathways (cruciform cleavage or degradation from a DSB followed by intrastrand annealing), the genetic requirements for the formation of the novel bands were investigated (Figure 4). Cruciforms are four-way junctions and, as such, structurally resemble Holliday junctions. The RuvABC and RecG proteins provide two pathways for Holliday junction resolution (Cromie et al., 2001) and therefore might be involved in processing cruciforms were they to form. However, neither the level of hairpins nor the level of inverted chromosome duplications decreased in *ruv* or *recG* mutants (as represented by the levels of 2 kb and 4 kb fragments in Figure 4, respectively). Furthermore, mutations in both *ruv* and *recG* or overexpression of RusA, another protein that can cleave four-way junctions (Sharples, 2001), had no effect on the level of hairpins or inverted chromosome duplications either (data not shown). These data argue that none of the RuvABC, RecG, or RusA pathways of Holliday junction resolution is responsible for the formation of the novel bands. On the other hand, the alternative pathway predicts that the hairpin end is generated following processing of a DNA DSB in the vicinity of the palindrome (Figure 1B). Therefore, the RecBCD complex and some associated exonucleases (such as RecJ and SbcB; also known as Exonuclease I) would be prime candidates to generate single-stranded tails permitting the formation of DNA hairpin ends. As shown in Figure 4A, RecB was essential for hairpin and inverted chromosome duplication formation. In addition, a *recD* or *sbcB* mutation severely reduced the formation of these two products. There was no effect of mutation in the *recJ* gene (data not shown). These data imply that the formation of gross chromosomal rearrangements in an *sbcDC recA* double-mutant strain containing the 246 bp palindrome is mainly initiated by a random DSB followed by degradation and intrastrand annealing at the palindromic locus (Figure 1B).

### An Induced DSB Increases the Number of Chromosomal Rearrangements

**Figure 5:**
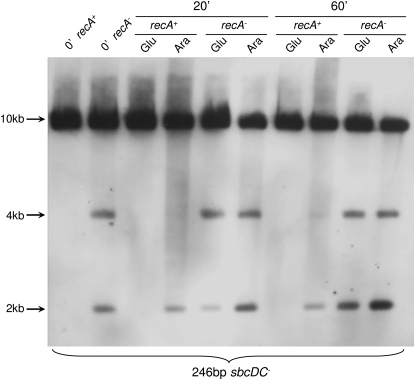
Induction of DSB Increased the Formation of the Novel DNA Fragments Cells were grown in LB complemented with 0.5% of glucose (Glu) until an OD_600nm_ of about 0.3 (Time 0′). Then, 0.2% of arabinose (Ara) was added to half of the culture, and samples for DNA extractions were taken after 20 and 60 min of incubation. The DNA was digested by the SacI enzyme, and the region surrounding the palindrome insertion place in *lacZ* was studied by Southern blot analysis using the origin-proximal probe in *lacZ*. The strains used for the analysis were the *sbcDC* mutant containing the arabinose inducible I-SceI system and the I-SceI restriction site 15 kb away from the 246 bp palindrome on the origin-distal side (DL3860) and the *sbcDC recA* mutant containing the arabinose-inducible I-SceI system and the I-SceI restriction site 15 kb away from the 246 bp palindrome on the origin-distal side (DL3870).

To investigate whether DSBs can stimulate these gross chromosomal rearrangements, the levels of hairpins and inverted chromosome duplications were investigated by Southern hybridization in *sbcDC* mutant strains in which a DSB could be specifically induced (Figure 5). The expression of the I-SceI enzyme was placed under the control of the P*_BAD_* promoter (expression of I-SceI was repressed in the presence of glucose and induced in the presence of arabinose), and a unique I-SceI cleavage site was introduced about 15 kb away from the 246 bp interrupted palindrome on the origin-distal side. In a *recA sbcDC* mutant strain, the level of hairpin formation on the origin-proximal side increased after induction of a DSB on the origin-distal side of the palindrome (in the presence of arabinose; Figure 5). Surprisingly, induction of a DSB in a *recA*^+^ *sbcDC^−^* strain also resulted in hairpin formation, although at a reduced level. By contrast to the stimulation of hairpin formation, no significant stimulation of inverted chromosome duplication was observed in either *recA*^−^ or *recA*^+^ cells. A similar stimulation of hairpin formation was observed on the origin-distal side of the palindrome when a DSB was induced on the origin-proximal side (data not shown).

### Visualization of the Chromosomal Rearrangements by Microscopy

**Figure 6:**
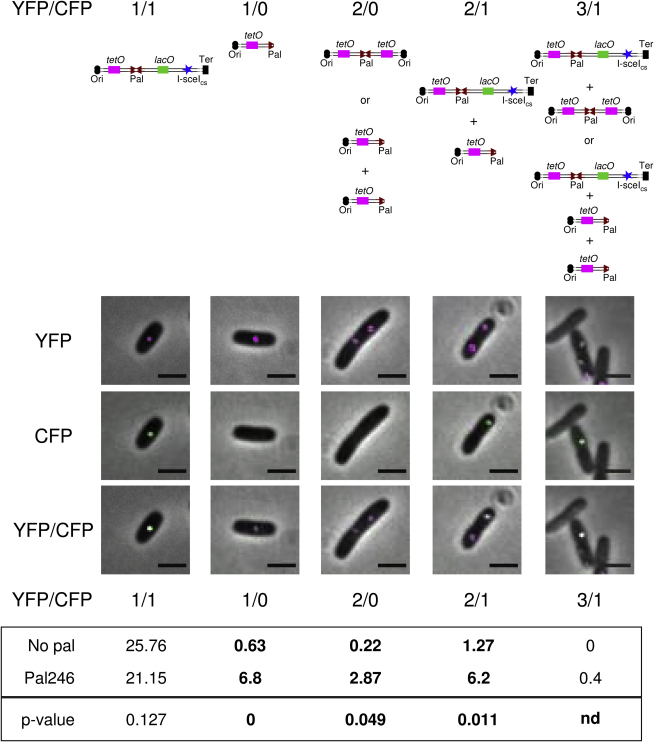
Visualization of the Chromosomal Rearrangements following DSB Cells were grown at 37°C in complemented M9 minimal medium supplemented with 0.5% of glucose until an OD_600nm_ of about 0.1, and then 0.2% of arabinose was added to the culture. After a further hour of growth, cells were visualized on a 1% agarose-coated slide by fluorescence microscopy. The strains used for the analysis were *sbcDC recA* mutants containing an arabinose-inducible I-SceI system, an I-SceI restriction site 15 kb away from *lacZ* on the origin-distal side, *tetO* and *lacO* arrays surrounding *lacZ*, and the plasmid pDL3196 expressing the LacI and TetR repressor proteins. DL4204 was palindrome free (No pal), and DL4206 contained a 246 bp palindrome in the lacZ gene (Pal246). Schematic representations of the region surrounding the palindrome insertion place in *lacZ* in a normal cell (YFP/CFP = 1/1) or after a chromosomal rearrangement indicate the location of the *tetO* array (magenta rectangle), the *lacO* array (green rectangle), and the I-SceI cutting site (blue star). In the microscopy pictures, the calibration bar indicates 2 μm. YFP fluorescence is indicated in magenta, whereas CFP fluorescence is indicated in green. YFP is an overlay of brightfield and YFP fluorescence. CFP is an overlay of brightfield and CFP fluorescence. YFP/CFP is an overlay of brightfield and YFP and CFP fluorescences. The mean of percentage of cells for each combination of YFP/CFP foci per cell was calculated from three independent experiments studying a total of 1300 cells by strain (see also Table S1). p values were calculated by two-sample t test (using Minitab). p values ≤ 0.05 were deemed to be statistically significant (indicated in bold) and could not be calculated when the three experiments performed on one strain had 0% of the tested YFP/CFP combination (nd).

In order to visualize the rearrangements following DSB induction in individual cells, the palindrome was flanked by sequences permitting the visualization of the DNA region by fluorescence microscopy. The 240 *tetO* sequences, on which TetR-YFP proteins can bind, were introduced 5.8 kb away from the palindrome on the origin-proximal side, and 240 *lacO* sequences, on which LacI-CFP proteins can bind, were introduced 4.8 kb away on the origin-distal side. TetR-YFP and LacI-CFP proteins were expressed from the plasmid pDL3196 (White et al., 2008). The distribution of YFP and CFP foci was investigated in an *sbcDC recA* double-mutant strain containing or not the 246 bp interrupted palindrome after induction of an I-SceI origin-distal DSB (Figure 6 and Table S1 available online). For a better quality of imaging, the microscopy was done following growth in minimal medium, in which the formation of hairpins and inverted chromosome duplications were also noticeable by Southern blot analysis (data not shown).

Following a DSB, the number of cells displaying one or two YFP foci alone or associations of two YFP foci and one CFP focus or of three YFP foci and one CFP focus was significantly higher in the strain containing the palindrome than in the strain that did not contain the palindrome (Figure 6 and Table S1). Figure 6 shows a representative cell for each of these categories, along with a cell with an undamaged chromosome, featuring one twin focus of YFP and CFP (1/1). Following induction of a DSB on the origin-distal side of the palindrome, the DNA would be degraded up to the palindrome, eliminating the *lacO* sequences and therefore the LacI-CFP binding. The single-stranded palindrome could then fold back on itself and become a hairpin. The origin-proximal *tetO* sequences would still be present, so a cell in which the palindrome became a hairpin after induction of a DSB on the origin-distal side would display a single YFP focus (Figure 6, 1/0) or two single YFP foci in cells containing two chromosomes (Figure 6, 2/0). Alternatively, the hairpin could be replicated in the absence of hairpin cleavage by the SbcCD complex forming an inverted chromosome duplication, which would be visualized as two single YFP foci in the cell (Figure 6, 2/0). Strikingly, after a DSB, the number of cells with one or two single YFP foci was more than 10 times higher in cells containing the palindrome. A cell containing an undamaged chromosome and a hairpin would display one twin YFP plus CFP focus pair and one single YFP focus (Figure 6, 2/1). Finally, a cell containing an undamaged chromosome and an inverted chromosome duplication, or two single hairpins, would display one twin YFP plus CFP focus pair and two single YFP foci (Figure 6, 3/1). After DSB induction, the number of cells with two or three YFP foci and one CFP focus was significantly higher in cells containing the palindrome. All together, after induction of a DSB, these four patterns of foci appeared in 16.3% of *sbcDC recA* double mutants containing the palindrome and only in 2.1% of *sbcDC recA* double mutants that do not contain the palindrome. These data show that the rearrangement events occurring after a DSB induction in an *sbcDC recA* double mutant containing the 246 bp palindrome can be visualized in individual cells using microscopy.

## Discussion

**Figure 7:**
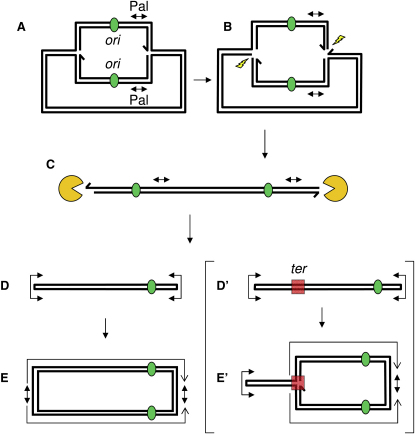
Degradation Mechanism for Formation of Inverted Duplications in Plasmids and Circular Chromosomes This mechanism derives from the model proposed by Lyu and collaborators (Lyu et al., 1999). In their model, Lyu and colleagues proposed that two DSBs in a replicating circular plasmid (A) would generate a linear molecule with two copies of the palindromic sequence (Pal). The present model suggests that these DSBs are generated at the replication forks (B), but any source of DSB could substitute for the proposed one, provided that there is one break on either side of the palindrome in the two replicated arms of the plasmid. Degradation from these breaks (C) will allow hairpin formation at each copy of the palindrome, generating a linear dumbbell structure (D), which, when replicated, generates the inverted duplication observed in bacterial and yeast plasmids (E). In a molecule such as the *E. coli* chromosome, containing a termination sequence for DNA replication (*ter*), this sequence will be present in the linear dumbbell (D′). Replication of the dumbbell will be arrested at *ter*, resulting in a partially replicated inverted chromosome duplication (E′). The origin of replication (*ori*) is denoted by a green oval, and the termination site (*ter*) is represented by a red square. The nucleases responsible for DNA degradation are represented by an orange symbol.

Three main hypotheses have been proposed to explain the formation of inverted chromosome duplications (Figure 1). The existence of a pathway involving intermolecular SSA of well-separated inverted repeats has been clearly established (Butler et al., 2002; Downing et al., 2008; VanHulle et al., 2007). Yet, the mechanism by which perfect and interrupted palindromes induce the formation of inverted chromosome duplication is more controversial. Several studies have suggested that the formation and resolution of a cruciform structure may lead to the formation of an inverted chromosome duplication (Coté and Lewis, 2008; Harada et al., 2009; Lin et al., 1997; Lobachev et al., 2002; Malagón and Aguilera, 1998; Narayanan et al., 2006). Studies providing the most direct evidence of this pathway have involved the use of plasmids in *E. coli* (Malagón and Aguilera, 1998) and *S. cerevisiae* (Coté and Lewis, 2008). Because plasmids are small and circular, it is possible to recover molecules with hairpins at both ends (a dumbbell structure) that, after replication, become inverted plasmid duplications. The existence of this dumbbell structure and its replication product has been demonstrated (Malagón and Aguilera, 1998). The simplest hypothesis to explain these observations is that the palindrome is extruded into a cruciform structure, which is resolved by a Holliday junction resolvase. Some support for this hypothesis has been obtained from the observation that Mus81 and RusA are able to stimulate the formation of the double-hairpin product and the inverted plasmid duplication in *S. cerevisiae* (Coté and Lewis, 2008). However, in *E. coli*, although dumbbell structures prepared in vitro can produce inverted chromosome duplications (Lin et al., 1997), Holliday junction resolvases are not implicated in the formation of these same products (Lyu et al., 1999; Malagón and Aguilera, 1998), and mismatched inverted repeats form heteroduplexes in a crossover-independent manner. These data argue strongly that cruciform resolution is not at the origin of dumbbell products and inverted plasmid duplications. In support of the existence of an alternative pathway for the formation of the dumbbell products, experiments have indicated that linear substrates containing terminal inverted repeats can result from DSBs during plasmid replication and can lead to the formation of a hairpin at each end of the molecule, resulting in the dumbbell intermediate and the inverted plasmid duplication (Figure 7; Lin et al., 2001; Lyu et al., 1999). Given that Mus81 cleaves replication forks more efficiently than Holliday junctions (Fricke et al., 2005), a version of the model of Lin and collaborators could account for the observations in *S. cerevisiae* (Coté and Lewis, 2008). In addition, RusA, which cleaves replication forks inefficiently (Bolt and Lloyd, 2002), substitutes poorly for Mus81 when overexpressed in *S. pombe* (Coté and Lewis, 2008). Further evidence in favor of the DNA degradation-hairpin pathway for formation of the inverted chromosome duplication has been obtained (Butler et al., 1996; Qin and Cohen, 2000; Tanaka et al., 2002; Tanaka et al., 2007; Tanaka and Yao, 2009). These experiments depend on the induction or stimulation of a DSB in the vicinity of the palindrome or inverted repeat to stimulate the formation of the inverted chromosome duplication. They argue for the existence of the hairpin pathway in the presence of induced breaks but have the weakness that they do not indicate the mechanism used in the spontaneous situation (i.e., in the absence of induced breaks). The present study demonstrates not only that induced DNA DSBs stimulate the formation of an inverted duplication in the *E. coli* chromosome, but also that the genetic requirements for the uninduced, spontaneous reaction are entirely consistent with the DNA degradation-hairpin formation mechanism and not with the cruciform cleavage mechanism.

### SbcCD and RecA Protect the Cell against the Formation of Inverted Chromosome Duplications

There are remarkable similarities between the pathways implicated in the formation of inverted chromosome duplications stimulated by perfect or interrupted palindromes in prokaryotes and eukaryotes. In *S. cerevisiae*, the Rad50/Mre11 protein complex plays a substantial role in preventing the formation of inverted chromosome duplications when inverted repeats are close enough to form hairpins that can be recognized by this complex (Coté and Lewis, 2008; Lobachev et al., 2002; Narayanan et al., 2006; Rattray et al., 2001, 2005). The work presented here demonstrates that the Rad50/Mre11 homolog SbcCD plays a similar role in the *E. coli* chromosome. In addition, loss of Rad51 in *S. pombe* stimulates the formation of inverted chromosome duplications at the centromere (Nakamura et al., 2008), and here, loss of the Rad51 homolog RecA similarly controls the reaction in the *E. coli* chromosome. This is expected in the DNA degradation-hairpin formation model because repair of the DSB by RecA-mediated recombination will prevent the opportunity for DNA degradation required for hairpin formation. Therefore, both RecA and SbcCD work to protect the genome integrity of *E. coli*. A previous study demonstrated that inverted duplications could form from a palindrome-containing plasmid introduced into a *recBC sbcBC* mutant (Malagón and Aguilera, 1998). However, understanding the involvement of SbcCD in that study was complicated by the fact that the plasmid could only replicate in cells lacking SbcCD. In the present study, the palindromes used are tolerated in the chromosomes of cells expressing SbcCD. The use of an 85 bp palindrome, which is tolerated in the presence of SbcCD even in the absence of RecA, demonstrates that formation of the inverted chromosome duplication is controlled by SbcCD. Of interest, this result indicates that the involvement of SbcCD in the cleavage of palindromes formed on the lagging strand template during DNA replication is separate from the role of SbcCD in the cleavage of hairpins formed as precursors to inverted chromosomal duplications. The former reaction is only detected with artificial palindromes longer than those found to occur naturally in the bacterial genome, whereas the latter reaction could be caused by any closely spaced inverted repeat, which generates a hairpin of size recognized by the SbcCD nuclease complex.

Several studies have linked defects in homologous recombination with an increased risk of specific chromosome rearrangement associated with a number of different cancers. For example, genes in the BRCA family control specific rearrangements detected in lymphomas and leukemias (Friedenson, 2007), and inactivation of BRCA2 leads to an elevation of chromosomal rearrangement in fetal liver cells (Yu et al., 2000). Of greatest interest, carriers of BRCA2 mutations have been shown to have a propensity for constitutional genome instability with inversions, duplications, and amplifications in the 9p23-24 chromosomal region (Savelyeva et al., 2001). Whether DNA misfolding plays a part in these events remains to be determined.

### DNA Degradation by RecBCD Is Required for the Spontaneous Events Leading to Inverted Chromosome Duplication

The genetic requirements for the rearrangement in the *sbcDC recA* permissive background were investigated in order to distinguish between the two models for palindrome-stimulated inverted chromosome duplication (cruciform cleavage or degradation and hairpin formation). If cruciform cleavage was at the origin of the rearrangements, the frequency of formation of the inverted chromosome duplication was expected to decrease in mutants defective in Holliday junction resolution. However, no reduction in inverted chromosome duplication in *ruv* and/or *recG* mutants was detected. Conversely, if DNA degradation and hairpin formation were at the origin of the rearrangements, the frequency of formation of the inverted chromosome duplication was expected to decrease in a *recB* mutant. Here, mutation in *recB* abolished the formation of hairpin and inverted chromosome duplication. The lack of a clear role for RecBCD in the plasmid-based assays (Bi and Liu, 1996; Malagón and Aguilera, 1998) is likely to have arisen from the small size of the substrates in which other nucleases are likely to be able to substitute and in which the voracious activity of RecBCD (particularly in the absence of *chi* sites) could easily lead to complete plasmid degradation. Nevertheless, a role for RecBCD has been detected when a substrate was specifically prepared to mimic the predicted DNA degradation-hairpin formation pathway for plasmids (Lin et al., 2001). In addition, the present work demonstrates that the hairpin formation is substantially reduced, but not totally eliminated, in *recD* and *sbcB* mutants. *recD* mutants are defective in one of the helicase motors of RecBCD and lack the nuclease activity (Dillingham and Kowalczykowski, 2008). The reduction observed in a *recD* mutant is consistent with DNA degradation being a major contributor to the formation of the inverted chromosome duplication. The involvement of SbcB (ExoI) is interesting given that the reaction is occurring in a *recA* mutant in which RecBCD-mediated DNA degradation is rampant. It is therefore likely that SbcB plays a direct role during the reaction. One possibility is that SbcB is required to degrade a 3′ single-strand tail linked to the hairpin prior to DNA synthesis. This role would bring the reaction observed in *E. coli* into line with the requirement for a 3′ single-stranded tail required for a similar reaction in *Streptomyces* (Qin and Cohen, 2000).

### Hairpin Formation Is Stimulated by a DNA DSB Induced in the Region of the Palindrome

This study demonstrates that induction of DSB at a single I-SceI restriction site 15 kb downstream of the palindrome resulted in an increase of hairpin formations in a *recA sbcDC* mutant. Moreover, in the presence of RecA, hairpin formation was detected following induction of a DSB. This second observation might reflect the fact that I-SceI endonuclease has the potential to cleave every chromosome at its recognition site, thereby removing intact template for repair, which results in DNA degradation up to the hairpin. DSB induction did not significantly stimulate the formation of inverted chromosome duplications in the presence or absence of RecA. It is currently not known why the induction of a DSB has relatively little effect on the formation of the inverted chromosome duplication. However, it may be that the substantial effect of cleaving all of the chromosomes at the I-SceI site inhibits DNA replication via an unknown mechanism.

### Visualization of DNA Rearrangement by Microscopy

Arrays of *lacO* and *tetO* operator sites, binding to LacI-CFP and TetR-YFP, respectively, were used to visualize the regions surrounding the palindrome in cells undergoing genome rearrangement. In order to increase the frequency of the events observed, DSBs were induced at the I-SceI site located 15 kb downstream of the palindrome. Under these conditions, *E. coli* containing the palindrome presented a significant increase in the frequency of cells with one single YFP focus and cells with two single YFP foci representative of hairpin and/or inverted chromosome duplications. Therefore, it is possible to visualize these events at the cellular level and to follow their progress in live cells.

### Conclusions

The present study shows that an interrupted palindrome in the *E. coli* chromosome stimulates spontaneous formation of an inverted chromosome duplication via a reaction that involves DNA degradation and hairpin formation following the formation of a spontaneous DSB. Furthermore, induction of a DSB adjacent to the palindrome increases the appearance of the hairpin intermediate. The palindrome used here is interrupted by a nonsymmetrical 24 bp sequence, which would be sufficient to prevent cruciform extrusion in vitro and does not behave as would be predicted were cruciform formation possible in vivo. Whether perfect palindromes form inverted chromosome duplications by a cruciform pathway in *E. coli* requires further investigation. However, this study confirms that the DNA degradation-hairpin formation pathway can explain spontaneous and DNA break-induced inverted chromosome duplication at an interrupted palindrome. This work provides a precedent to argue that spontaneous events occurring in mammalian cells may follow this same pathway, as it was previously demonstrated that induced DNA breaks stimulate inverted chromosome duplication by the DNA degradation-hairpin formation pathway (Tanaka et al., 2002; Tanaka et al., 2007). Finally, the present data imply that hairpin cleavage (SbcCD) and homologous recombination (RecA) both act to prevent this rearrangement, an observation that has been more difficult to establish in eukaryotic systems given that the SbcCD homolog Rad50/Mre11 is implicated in both hairpin cleavage and recombination.

## Experimental Procedures

### Plasmids, Bacterial Strains, Primers, Media, and DNA Techniques

DNA techniques, media, oligonucleotides, plasmids, and bacterial strains are described in Supplemental Experimental Procedures.

### Gel Electrophoresis and Southern Blotting

Cultures were grown at 37°C in LB broth until midexponential phase (between 0.6 and 1 OD_600nm_). Samples for gel electrophoresis were prepared using 2 OD_600nm_ of culture according to Michel and collaborators (Michel et al., 1997). DNA embedded in agarose plugs was digested at 37°C for 4.5 hr by 50 units of restriction enzyme (New England Biolabs).

Plugs containing digested DNA were loaded onto 0.8% high-strength agarose gels (AquaPor ES) in 1 × TBE. Gels were run overnight in 1 × TBE at 55 V and 4°C. PFGE were carried out as described by Eykelenboom and collaborators (Eykelenboom et al., 2008). Standard capillary transfer methods were used as described by Sambrook and collaborators (Sambrook et al., 1989). DNA was fixed to a positively charged nylon membrane by UV crosslinking. Generation of digoxigenin-labeled DNA probes (*lacZ* or *lacI*) was carried out using the PCR DIG probe synthesis kit (Roche), using primers DigLacZF and DigLacZR or DigLacIF and DigLacIR, respectively.

Plugs containing digested DNA were analyzed by 2D native/denaturing electrophoresis gel as described by Oh and collaborators (Oh et al., 2009).

### Fluorescence Microscopy

Cultures were grown at 37°C in complemented M9 minimal medium supplemented with 0.5% of glucose. Of note, cells were grown in the presence of 100 mg/l of ampicillin as selection for the pDL3196 plasmid and 100 μg/l of anhydrotetraclycine to prevent operator-bound TetR-YFP from blocking replication (Possoz et al., 2006). At ∼0.1 OD_600nm_, the culture was split in two, and 0.2% of arabinose was added to half of the culture. After an hour of growth, 10 μl of cells were placed onto a 1% agarose-coated slide. A total of 1300 cells per strain were analyzed from three independent experiments. Percentages of YFP/CFP foci for each strain were calculated, and p values were assessed by one-way unstacked ANOVA.

## Supplemental Information

Document S1. Supplemental Experimental Procedures and One Table
