# Supplementary material for: E. coli SbcCD and RecA Control Chromosomal Rearrangement Induced by an Interrupted Palindrome
Source: Mol Cell. 2010 Jul 9;39(1-2):59–70. doi: 10.1016/j.molcel.2010.06.011 (PMC3315005; doi:10.1016/j.molcel.2010.06.011)
Supplement: Document S1. Supplemental Experimental Procedures and One Table [file mmc1.pdf]

Molecular Cell, *Volume 39*

Supplemental Information

E. coli SbcCD and RecA Control Chromosomal Rearrangement Induced by an Interrupted Palindrome

Elise Darmon, John K. Eykelenboom, Frédéric Lincker, Lucy H. Jones, Martin White, Ewa Okely, John K. Blackwood, and David R. Leach

Supplemental Experimental Procedures

DNA techniques

Procedures for DNA purification, agarose gel electrophoresis and transformation of competent *E. coli* cells were carried out as described by Sambrook *et al.* (Sambrook et al., 1989). Enzymes were from New England Biolabs, Roche Molecular Biochemicals or Promega Life Science. Reactions were carried out according to manufacturer's instructions.

The pTOFmhpREcoRI plasmid was used to introduce an EcoRI cleavage site into the *mhpR* gene. Two amplified fragments in the *mhpR* region were ligated, after PCR-mediated coupling, into the chromosomal integration and excision plasmid pTOF24. The upstream fragment of 463bp was amplified from *E. coli* MG1655 using primers 2DEcoRI-F1 and 2DEcoRI-R1. The downstream fragment of 430bp was amplified from *E. coli* MG1655 using primers 2DEcoRI-F2 and 2DEcoRI-R2. Notably, primers 2DEcoRI-R1 and 2DEcoRI-F2 permitted the introduction of a unique EcoRI restriction site at the centre of the PCR-mediated coupling of these two PCR fragments. This 865bp final PCR product was cloned between the PstI and SalI sites of plasmid pTOF24, resulting in the kanamycin sensitive pTOFmhpREcoRI plasmid.

The pDL2038 plasmid was used to introduce an 85bp palindrome into the *lacZ* gene. A 513bp fragment containing the 85bp palindrome was amplified by PCR, using primers pal-CF2 and pal-CR1 and the KOD polymerase (Novagen), from the bacteriophage λ containing del85 isolated by Pinder and collaborators (Pinder et al., 1998). The 85bp palindrome was excised by digestion with EcoRI and cloned into the MfeI restriction site of the chromosomal integration and excision plasmid pLacD1, resulting in the kanamycin sensitive pDL2038 plasmid. The presence of the palindrome in the plasmid was verified by PCR using primers lac-SF1 and seq-SR1.

The pTOF<cynXhomol> plasmid was used to remove the *tetO* array [240x*tetO*(Gm^R)] from the *cynX* gene. A fragment of 974bp was amplified by PCR from the *E. coli* chromosome using primers TetO-CF1 and TetO-CR2. This fragment was digested by PstI and NheI restriction enzymes and cloned between the PstI and XbaI sites of the chromosomal integration and excision plasmid pTOF24, resulting in the kanamycin sensitive pTOF<cynXhomol> plasmid.

The pTOFruvC plasmid was used to introduce an in-frame deletion of the *ruvC* gene. Two amplified fragments in the *ruvC* region were ligated, after PCR-mediated coupling, into the pTOF24 plasmid. The 429bp-upstream fragment and the 439bp-downstream fragment were amplified from *E. coli* MG1655 using primer pairs ruvC-KO-F1/ruvC-KO-R1 and ruvC-KO-F2/ruvC-KO-R2, respectively. The 844bp PCR-mediated coupling of these two fragments was cloned between the PstI and SalI sites of plasmid pTOF24, resulting in the kanamycin sensitive pTOFruvC plasmid.

The pTOFrecD plasmid was used to introduce an in-frame deletion of the *recD* gene. Two amplified fragments in the *recD* region were ligated, after PCR-mediated coupling, into the pTOF24 plasmid. The 458bp-upstream fragment and the 472bp-downstream fragment were amplified from *E. coli* MG1655 using primer pairs recD-KO-F1/recD-KO-R1 and recD-KO-

F2/recD-KO-R2, respectively. The 906bp PCR-mediated coupling of these two fragments was cloned between the PstI and SalI sites of plasmid pTOF24, resulting in the kanamycin sensitive pTOFrecD plasmid.

The pTOFsbcB plasmid was used to introduce an in-frame deletion of the s*bcB* gene. Two amplified fragments in the s*bcB* region were ligated, after PCR-mediated coupling, into the pTOF24 plasmid. The 466bp-upstream fragment and the 438bp-downstream fragment were amplified from *E. coli* MG1655 using primer pairs sbcB-KO-F1/sbcB-KO-R1 and sbcB-KO-F2/sbcB-KO-R2, respectively. The 880bp PCR-mediated coupling of these two fragments was cloned between the PstI and SalI sites of plasmid pTOF24, resulting in the kanamycin sensitive pTOFsbcB plasmid.

The pTOFrecB plasmid was used to introduce an in-frame deletion of the *recB* gene. Two amplified fragments in the *recB* region were ligated, after PCR-mediated coupling, into the pTOF24 plasmid. The 446bp-upstream fragment and the 460bp-downstream fragment were amplified from *E. coli* MG1655 using primer pairs recB-KO-F1/recB-KO-R1 and recB-KO-F2/recB-KO-R2, respectively. The 882bp PCR-mediated coupling of these two fragments was cloned between the PstI and SalI sites of plasmid pTOF24, resulting in the kanamycin sensitive pTOFrecB plasmid.

The pYaiOIsceI plasmid was used to introduce an I-SceI cleavage site into the *yaiO* gene. Two amplified fragments in the *yaiO* region were ligated, after PCR-mediated coupling, into the pTOF24 plasmid. The 436bp-upstream fragment and the 402bp-downstream fragment were amplified from *E. coli* MG1655 using primer pairs yaiO1/yaiO2 and yaiO3/yaiO4, respectively. Notably, primers yaiO2 and yaiO3 added an I-SceI restriction site between the two fragments. The 820bp PCR-mediated coupling of these two fragments was cloned between

the PstI and SalI sites of plasmid pTOF24, resulting in the kanamycin sensitive pYaiOIsceI plasmid.

In order to introduce a mutation using a derivative of the pTOF24 or pLacD1 plasmid, the specific thermosensitive plasmid was introduced into the *E. coli* chromosome of the original strain with selection for chloramphenicol resistance. Then, an allele replacement by chromosomal integration and excision was carried out as described by Merlin and collaborators (Merlin et al., 2002). Correct insertion of the mutation was verified by PCR using external primers (or primers Ex-test-F and Ex-test-R for a palindrome). If applicable, the insertion of a restriction site was checked by restriction of the PCR product or deletions were verified by UV sensitivity test.

Specific mutations were transferred into the desired receiving strains using the P1 transduction technique with selection for the appropriate antibiotic resistance (Miller J.H., 1992). When applicable, the presence of the palindrome was verified by PCR using primers Ex-test-F and Ex-test-R. In order to do the P1 transduction into the MG1655 *lacZ*χ- *lacI*^{q} *lacZ*::pal246 *cynX*::Gm^{r} Δ*sbcDC mhpR*(EcoRI) Δ*recB* (DL4116) strain, the pAM-RecBCD+ plasmid was transiently used to make this strain recombination proficient.

Fluorescence microscopy

Images were acquired at a resolution of 0.129µm per pixel using a Zeiss Axiovert 200 fluorescence microscope equipped with a Photometrics cool-SNAP HQ CCD camera. Stacks of optical section images of CFP and YFP fluorescence were collected and deconvolved using the Autovisualize + Autodeblur program (3D adaptative PSF (blind) deconvolution), then analysed and pseudocoloured using the MetaMorph 6-3r2 program (Molecular Devices).

Plasmids, bacterial strains, primers and media

Bacteria were grown at 37ºC under agitation in either LB (1% Bactotryptone, 0.5% Bacto yeast extract, 0.5% NaCl and 2mM NaOH) or M9 medium (50mM Na_2HPO_4, 22mM KH_2PO_4, 8.5mM NaCl and 2mM NH_4Cl, supplemented with 0.02mM $CaCl_2$, 1mM $MgSO_4$). Antibiotics were used at the following concentrations: ampicillin (Amp), 100mg/l; chloramphenicol (Cm), 50mg/l; gentamicin (Gm), 10mg/l and kanamycin (Km), 50mg/l. IPTG was used at 0.28mM. Sucrose was used a 5%, glucose at 0.5% and arabinose at 0.2%.

Plasmids, bacterial strains and primers. Ts indicates that the plasmid has a temperature-sensitive origin of replication. *lacZχ-* indicates that a *chi* sequence situated at 2230bp in the *lacZ* gene was removed. Restriction sites used for cloning are underlined and sequences required for the PCR-mediated coupling are indicated in bold letters.

**Plasmids**	**Relevant properties**	**References or construction**
pAM-recBCD+	pAM34 derivative plasmid containing the *recBCD* genes under the control of a constitutive promoter; IPTG, Amp^R	Gift from Benedicte Michel
pDL2038	pLacD1 derivative containing an 85bp palindrome; Cm^r, Ts, Suc^s	This work
pDL2736	pTOF24 derivative containing two fused PCR fragments from the *proA* region, separated by an I-SceI restriction site, in the place of the *aph* gene; Cm^r, Ts, Suc^s	(Eykelenboom et al., 2008)
pDL2755	pTOF24 derivative containing two fused PCR fragments from the *tsx* region, separated by an I-SceI restriction site, in the place of the *aph* gene; Cm^r, Ts, Suc^s	(Eykelenboom et al., 2008)
pDL2774	pLacD1 derivative containing a 246bp palindrome; Cm^r, Ts, Suc^s	(Eykelenboom et al., 2008)
pDL3196	*lacI-cfp* (cerulean) and *tetR-yfp* under a weak constitutive promoter (P_{ftsKi}); Amp^R	(White et al., 2008)

pLacD1	pTOF24 derivative containing two fused PCR fragments from the *lacZ* region, the L8 mutation and in which the *Bsa*I, *Mfe*I, and *Bbs*I restriction sites were moved to the beginning of the *lacZ* gene; Cm^r, Ts, Suc^s	(Zahra et al., 2007)
pTOF24	pSC101-based vector; *repA*(Ts) with a *sacB* gene conferring sucrose sensitivity and *aph* from pUC4K; Cm^r, Km^r, Ts, Suc^s	(Merlin et al., 2002)
pTOF<cynXhomol>	pTOF24 derivative containing a PCR fragment from the *cynX* region in the place of the *aph* gene; Cm^r, Ts, Suc^s	This work
pTOFmhpREcoRI	pTOF24 derivative containing two fused PCR fragments, from the *mhpR* region, separated by an EcoRI restriction site, in the place of the *aph* gene; Cm^r, Ts, Suc^s	This work
pTOFrecB	pTOF24 derivative containing two fused PCR fragments, homologous to upstream and downstream regions of *recB*, in the place of the *aph* gene; Cm^r, Ts, Suc^s	This work
pTOFrecD	pTOF24 derivative containing two fused PCR fragments, homologous to upstream and downstream regions of *recD*, in the place of the *aph* gene; Cm^r, Ts, Suc^s	This work
pTOFruvAB	pTOF24 derivative containing two fused PCR fragments, homologous to upstream and downstream regions of the *ruvAB* operon, in the place of the *aph* gene; Cm^r, Ts, Suc^s	(Eykelenboom et al., 2008)
pTOFruvC	pTOF24 derivative containing two fused PCR fragments, homologous to upstream and downstream regions of *ruvC*, in the place of the *aph* gene; Cm^r, Ts, Suc^s	This work
pTOFsbcB	pTOF24 derivative containing two fused PCR fragments, homologous to upstream and downstream regions of *sbcB*, in the place of the *aph* gene; Cm^r, Ts, Suc^s	This work
pTOFsbcDC	pTOF24 derivative containing two fused PCR fragments, homologous to upstream and downstream regions of *sbcDC* operon, in the place of the *aph* gene; Cm^r, Ts, Suc^s	(Darmon et al., 2007)
pYaiOIsceI	pTOF24 derivative containing two fused PCR fragments from the *yaiO* region, separated by an I-SceI restriction site, in the place of the *aph* gene; Cm^r, Ts, Suc^s	This work

**Strains**		
E. coli		
BW27784	*lacI*^{q} *rrnB3*Δ*lacZ4787* *hsdR514* DE(*araBAD*)*567* DE(*rhaBAD*)*568* DE(*araFGH*) Φ(Δ*araEp* P$_{CP18}$-*araE*)	(Khlebnikov et al., 2001)
DB1318	*recD*1014 *hsdR*$_{2}$ *Zgq*-202::Tn10 *recA*::*cat*	(Wertman et al., 1986)
DL1777	MG1655 *lacZ*χ- *lacI*^{q}	(Eykelenboom et al., 2008)
DL2006	BW27784 *lacZ*χ- *lacI*^{q} P$_{araBAD}$-*sbcDC* *lacZ*::pal246 *cynX*::Gm^{r}	(Eykelenboom et al., 2008)
DL2151	MG1655 *lacZ*χ- *lacI*^{q} Δ*sbcDC*	(Eykelenboom et al., 2008)
DL2792	BW27784 P$_{araBAD}$-*sbcDC* *cynX*::GmR *tsx*::I-SceI$_{cs}$ *proA*::I-SceI$_{cs}$	(Eykelenboom et al., 2008)
DL2849	BW27784 P$_{araBAD}$-*sbcDC* *cynX*::GmR *tsx*::I-SceI$_{cs}$ *proA*::I-SceI$_{cs}$ *lacZ*::I-SceI$_{cs}$	(Eykelenboom et al., 2008)
DL2874	MG1655 *lacZ*χ- *lacI*^{q} *lacZ*::pal246 *cynX*::Gm^{r} Δ*sbcDC*	(Eykelenboom et al., 2008)
DL2894	BW27784 *lacZ*$^{+}$ *lacZ*χ- *lacI*^{q} *cynX*::[240x*tetO*(Gm^{R})] *araB*::P$_{araBAD}$-*I-sceI*	(White et al., 2008)
DL2988	BW27784 *lacZ*$^{+}$ *lacZ*χ- *lacI*^{q} *araB*::P$_{araBAD}$-*I-sceI*	DL2894 mutated using pTOF<cynXhomol>
DL3279	BW27784 *lacZ*$^{+}$ *lacZ*χ- Δ*lacI* *mhpA*::χχχ *lacZY*::χχχ *araB*::P$_{araBAD}$-*I-sceI* *cynX*::[240x*tetO*(Gm^{R})] *mhpC*::[240x*lacO*(Km^{R})]	(White et al., 2008)
DL3325	MG1655 *lacZ*χ- *lacI*^{q} Δ*sbcDC* *mhpR*(EcoRI)	DL2151 mutated using pTOFmhpREcoRI
DL3326	MG1655 *lacZ*χ- *lacI*^{q} *lacZ*::pal246 *cynX*::Gm^{r} Δ*sbcDC* *mhpR*(EcoRI)	DL2874 mutated using

		pTOFmhpREcoRI
DL3395	MG1655 *lacZ*χ- *lacI*^{q} Δ*sbcDC mhpR*(EcoRI) *recA*::Cm	DL3325 mutated by P1 from DB1318
DL3396	MG1655 *lacZ*χ- *lacI*^{q} *lacZ*::pal246 *cynX*::Gm^{r} Δ*sbcDC mhpR*(EcoRI) *recA*::Cm	DL3326 mutated by P1 from DB1318
DL3752	MG1655 *lacZ*χ- *lacI*^{q} *lacZ*::pal246 *cynX*::Gm^{r} Δ*sbcDC mhpR*(EcoRI) Δ*ruvAB*	DL3326 mutated using pTOFruvAB
DL3753	MG1655 *lacZ*χ- *lacI*^{q} *lacZ*::pal246 *cynX*::Gm^{r} Δ*sbcDC mhpR*(EcoRI) Δ*ruvAB recA*::*cat*	DL3752 mutated by P1 from DB1318
DL3754	MG1655 *lacZ*χ- *lacI*^{q} *lacZ*::pal246 *cynX*::Gm^{r} Δ*sbcDC mhpR*(EcoRI) Δ*ruvC*	DL3326 mutated using pTOFruvC
DL3755	MG1655 *lacZ*χ- *lacI*^{q} *lacZ*::pal246 *cynX*::Gm^{r} Δ*sbcDC mhpR*(EcoRI) Δ*ruvC recA*::*cat*	DL3754 mutated by P1 from DB1318
DL3804	MG1655 *lacZ*χ- *lacI*^{q} *lacZ*::pal246 *cynX*::Gm^{r} Δ*sbcDC mhpR*(EcoRI) Δ*recG263*::*kan*	DL3326 mutated by P1 from N3793
DL3810	MG1655 *lacZ*χ- *lacI*^{q} *lacZ*::pal246 *cynX*::Gm^{r} Δ*sbcDC mhpR*(EcoRI) Δ*recG263*::*kan recA*::*cat*	DL3804 mutated by P1 from DB1318
DL3812	MG1655 *lacZ*χ- *lacI*^{q} *lacZ*::pal246 *cynX*::Gm^{r} Δ*sbcDC mhpR*(EcoRI) Δs*bcB*	DL3326 mutated using pTOFsbcB
DL3827	MG1655 *lacZ*χ- *lacI*^{q} *lacZ*::pal246 *cynX*::Gm^{r} Δ*sbcDC mhpR*(EcoRI) Δs*bcB recA*::*cat*	DL3812 mutated by P1 from DB1318
DL3828	BW27784 *lacZ*$^{+}$ *lacZ*χ- *lacI*^{q} *araB*::P$_{araBAD}$-*I-sceI* Δ*sbcDC*	DL2988

		mutated using pTOFsbcDC
DL3835	MG1655 *lacZ*χ- *lacI*^{q} Δ*sbcDC lacZ*::pal85	DL2151 mutated using pDL2038
DL3845	BW27784 *lacZ*$^+$ *lacZ*χ- *lacI*^{q} *araB*::P$_{araBAD}$-*I-sceI* Δ*sbcDC* *yaiO*::I-SceI$_{cs}$	DL3828 mutated using pYaiOIsceI
DL3846	MG1655 *lacZ*χ- *lacI*^{q} *lacZ*::pal85	DL1777 mutated using pDL2038
DL3853	BW27784 *lacZ*$^+$ *lacZ*χ- Δ*lacI* *mhpA*::χχχ *lacZY*::χχχ *araB*::P$_{araBAD}$-*I-sceI* *cynX*::[240x*tetO*(Gm^R)] *mhpC*::[240x*lacO*(Km^R)] Δ*sbcDC*	DL3279 mutated using pTOFsbcDC
DL3856	MG1655 *lacZ*χ- *lacI*^{q} Δ*sbcDC lacZ*::pal85 *recA*::Cm	DL3835 mutated by P1 from DB1318
DL3857	MG1655 *lacZ*χ- *lacI*^{q} *lacZ*::pal85 *recA*::Cm	DL3846 mutated by P1 from DB1318
DL3860	BW27784 *lacZ*$^+$ *lacZ*χ- *lacI*^{q} *araB*::P$_{araBAD}$-*I-sceI* Δ*sbcDC* *yaiO*::I-SceI$_{cs}$ *lacZ*::pal246 *cynX*::Gm^r	DL3845 mutated by P1 from DL2006
DL3867	MG1655 *lacZ*χ- *lacI*^{q} *lacZ*::pal246 *cynX*::Gm^r Δ*sbcDC* *mhpR*(EcoRI) Δ*recD*	DL3326 mutated using pTOFrecD
DL3870	BW27784 *lacZ*$^+$ *lacZ*χ- *lacI*^{q} *araB*::P$_{araBAD}$-*I-sceI* Δ*sbcDC* *yaiO*::I-SceI$_{cs}$ *lacZ*::pal246 *cynX*::Gm^r *recA*::Cm	DL3860 mutated by P1 from DB1318
DL3876	MG1655 *lacZ*χ- *lacI*^{q} *lacZ*::pal246 *cynX*::Gm^r Δ*sbcDC* *mhpR*(EcoRI) Δ*recD recA*::*cat*	DL3867 mutated by P1 from DB1318
DL4116	MG1655 *lacZ*χ- *lacI*^{q} *lacZ*::pal246 *cynX*::Gm^r Δ*sbcDC* *mhpR*(EcoRI) Δ*recB*	DL3326 mutated using pTOFrecB

DL4136	MG1655 *lacZχ- lacI*^{q} *lacZ*::pal246 *cynX*::Gm^{r} Δ*sbcDC mhpR*(EcoRI) Δ*recB recA*::*cat*	DL4116 mutated by P1 from DB1318
DL4200	BW27784 *lacZ*$^{+}$ *lacZχ-* Δ*lacI mhpA*::χχχ *lacZY*::χχχ *araB*::P$_{araBAD}$-*I-sceI* *cynX*::[240x*tetO*(Gm^{R})] *mhpC*::[240x*lacO*(Km^{R})] Δ*sbcDC yaiO*::I-SceI$_{cs}$	DL3853 mutated using pYaiOIsceI
DL4204	BW27784 *lacZ*$^{+}$ *lacZχ-* Δ*lacI mhpA*::χχχ *lacZY*::χχχ *araB*::P$_{araBAD}$-*I-sceI* *cynX*::[240x*tetO*(Gm^{R})] *mhpC*::[240x*lacO*(Km^{R})] Δ*sbcDC yaiO*::I-SceI$_{cs}$ *recA*::*cat*	DL4200 mutated by P1 from DB1318
DL4205	BW27784 *lacZχ-* Δ*lacI mhpA*::χχχ *lacZY*::χχχ *araB*::P$_{araBAD}$-*I-sceI* *cynX*::[240x*tetO*(Gm^{R})] *mhpC*::[240x*lacO*(Km^{R})] Δ*sbcDC yaiO*::I-SceI$_{cs}$ *lacZ*::pal246	DL4200 mutated using pDL2774
DL4206	BW27784 *lacZχ-* Δ*lacI mhpA*::χχχ *lacZY*::χχχ *araB*::P$_{araBAD}$-*I-sceI* *cynX*::[240x*tetO*(Gm^{R})] *mhpC*::[240x*lacO*(Km^{R})] Δ*sbcDC yaiO*::I-SceI$_{cs}$ *lacZ*::pal246 *recA*::*cat*	DL4205 mutated by P1 from DB1318
DL4421	MG1655 *lacZχ- lacI*^{q} Δ*sbcDC mhpR*(EcoRI) *tsx*::I-SceI$_{cs}$	DL3325 mutated using pDL2755
DL4422	MG1655 *lacZχ- lacI*^{q} *lacZ*::pal246 *cynX*::Gm^{r} Δ*sbcDC mhpR*(EcoRI) *tsx*::I-SceI$_{cs}$	DL3326 mutated using pDL2755
DL4461	MG1655 *lacZχ- lacI*^{q} Δ*sbcDC mhpR*(EcoRI) *tsx*::I-SceI$_{cs}$ *proA*::I-SceI$_{cs}$	DL4421 mutated using pDL2736
DL4466	MG1655 *lacZχ- lacI*^{q} *lacZ*::pal246 *cynX*::Gm^{r} Δ*sbcDC mhpR*(EcoRI) *tsx*::I-SceI$_{cs}$ *proA*::I-SceI$_{cs}$	DL4422 mutated using pDL2736
DL4480	MG1655 *lacZχ- lacI*^{q} Δ*sbcDC mhpR*(EcoRI) *tsx*::I-SceI$_{cs}$ *proA*::I-SceI$_{cs}$ *recA*::*cat*	DL4461 mutated by P1 from DB1318
DL4481	MG1655 *lacZχ- lacI*^{q} *lacZ*::pal246 *cynX*::Gm^{r} Δ*sbcDC mhpR*(EcoRI) *tsx*::I-SceI$_{cs}$ *proA*::I-SceI$_{cs}$ *recA*::*cat*	DL4466 mutated by P1 from DB1318

MG1655	F^- λ^- *ilvG*$^-$ *rfb-50 rph-1*	(Blattner et al., 1997)
N3793	AB1157 Δ*recG263*::*kan*	(Mahdi et al., 1996)
**Primers**	**5´- 3´**	
2DEcoRI-F1	AAAAAGTCGACAAACAAGCAGCGCACATTC	This work
2DEcoRI-R1	**ATCGGAGGGGAA**TTCATTTTGTCCGAGTCGTGAGG	This work
2DEcoRI-F2	**GGACAAAATGAA**TTCCCCTCCGATGATAGTTTTCG	This work
2DEcoR1-R2	AAAAACTGCAGCTGTCCGTAACCCTCTTTGC	This work
DigLacZF	CTGGCGTAATAGCGAAGAGG	This work
DigLacZR	CATGACCTGACCATGCAGAG	This work
DigLacIF	GAAAACGCGGGAAAAAGTG	This work
DigLacIR	GCATTAATGAATCGGCCAAC	This work
Ex-Test-F	TTATGCTTCCGGCTCGTATG	(Eykelenboom et al., 2008)
Ex-Test-R	GGCGATTAAGTTGGGTAACG	(Eykelenboom et al., 2008)
lac-SF1	AGTGGGATACGACGATACCG	This work
pal-CF2	ATACCCAGATTGCGAACACC	This work
pal-CR1	ACAACCTGACCCAGCAAAAG	This work
recB-KO-F1	AAAAACTGCAGTACAAGGCGTTTTTCCCAAC	This work
recB-KO-R1	**ATCCATCAGGGC**GCGCAAAGGATCTAGTGTCTCG	This work
recB-KO-F2	**GATCCTTTGCGC**GCCCTGATGGATGAGATGTTTG	This work
recB-KO-R2	AAAAAGTCGACCAATGGCATGATTCACTTCG	This work
recD-KO-F1	AAAAACTGCAGGTTAATCCGCCAGTTTGACC	This work
recD-KO-R1	**CAATTACGTTTA**TTTCATTACGCCTCCTCCAG	This work
recD-KO-F2	**GGCGTAATGAAA**TAAACGTAATTGCCGGATGC	This work
recD-KO-R2	AAAAAGTCGACGGAGCAGCAAGGTATTCTGG	This work
ruvC-KO-F1	AAAAACTGCAGATGGTTCCGTTGCCTATCTG	This work
ruvC-KO-R1	**TCGCATTCTGAC**TAATAGCCATCACGCGTCTC	This work
ruvC-KO-F2	**TGATGGCTATTA**GTCAGAATGCGATGCAGATG	This work
ruvC-KO-R2	AAAAAGTCGACGGCTGACAGAACGACAAAAAC	This work
sbcB-KO-F1	AAAAACTGCAGAACCCGTCATCAGCTTTGTC	This work

sbcB-KO-R1	**CCGCGTACTGCC**ATTGTTGCTTACCGTCATTCATC	This work
sbcB-KO-F2	**GTAAGCAACAAT**GGCAGTACGCGGAAGAGATTG	This work
sbcB-KO-R2	AAAAAGTCGACGCTGGATTGGCCTTGTATTT	This work
seq-SR1	GTGCTGCAAGGCATTAAGT	This work
TetO-CF1	AAAAAGCTAGCAAATATCTGCCGACCAAACC	(White et al., 2008)
TetO-CR2	AAAAACTGCAGCCCAGACCTAACCCACACAC	(White et al., 2008)
yaiO1	AAAAACTGCAGCGAATTATTTCCCCGAACAC	This work
yaiO2	**ATTACCCTGTTATCCCTA**TTATCCGCAAAGGCAATACC	This work
yaiO3	**TAGGGATAACAGGGTAAT**TACGCCGGTCTTTGCCCG	This work
yaiO4	AAAAAGTCGACTCAGTCAGCGGTTGAATACG	This work

Table S1: Quantification of chromosomal rearrangements following induction of DSBs (related to Figure 6). Percentage of cells for each combination of YFP/CFP foci per cell. Cells containing other combination of foci than the ones indicated in this table were not found in the studied population. Three independent experiments (Exp. 1, Exp. 2 and Exp. 3) were performed to study a total of 1,300 cells by strain. The means of these experiments are indicated followed by 95% confidence intervals (Mean ± 95%) and p-values calculated by two-sample t-test (using Minitab). p-values ≤ 0.05 were deemed to be statistically significant (indicated in bold) and could not be calculated when the three experiments performed on one strain had 0% of the tested YFP/CFP combination (nd). The strains used for the analysis were the *sbcDC recA* mutant containing the arabinose inducible I-SceI system, the I-SceI restriction site 15kb away from *lacZ* on the origin-distal side and the *tetO* and *lacO* arrays surrounding *lacZ* (DL4204) carrying the plasmid pDL3196 (No pal) and the *sbcDC recA* mutant containing the arabinose inducible I-SceI system, the I-SceI restriction site 15kb away from the 246bp palindrome on the origin-distal side and the *tetO* and *lacO* arrays surrounding the palindrome (DL4206) carrying the plasmid pDL3196 (Pal246).

Table S1.

	YFP	CFP	Exp. 1	Exp. 2	Exp. 3	Mean ± 95%	p-value
No pal<br>Pal246	0<br>0	0<br>0	27<br>17.59	41.55<br>22.22	41.67<br>27.9	36.74 ± 20.95<br>22.57 ± 12.82	0.089
No pal<br>Pal246	0<br>0	1<br>1	0.87<br>0.87	0.44<br>0.22	0<br>0.36	0.44 ± 1.08<br>0.48 ± 0.85	0.893
No pal<br>Pal246	0<br>0	2<br>2	0<br>0.35	0<br>0	0<br>0.36	0 ± 0<br>0.23 ± 0.51	nd
**No pal<br>Pal246**	**1<br>1**	**0<br>0**	**0.87<br>6.62**	**0.67<br>6.89**	**0.36<br>6.88**	**0.63 ± 0.63<br>6.8 ± 0.38**	**0**
No pal<br>Pal246	1<br>1	1<br>1	24.04<br>21.25	29.33<br>20.44	23.91<br>21.74	25.76 ± 7.68<br>21.15 ± 1.62	0.127
No pal<br>Pal246	1<br>1	2<br>2	0.52<br>0	0.67<br>1.33	0.72<br>0.72	0.64 ± 0.26<br>0.69 ± 1.66	0.913
**No pal<br>Pal246**	**2<br>2**	**0<br>0**	**0<br>2.44**	**0.67<br>4**	**0<br>2.17**	**0.22 ± 0.96<br>2.87 ± 2.45**	**0.049**
**No pal<br>Pal246**	**2<br>2**	**1<br>1**	**1.57<br>4.88**	**0.44<br>7.56**	**1.81<br>6.16**	**1.27 ± 1.81<br>6.2 ± 3.33**	**0.011**
No pal<br>Pal246	2<br>2	2<br>2	41.46<br>43.03	24.22<br>32.89	28.26<br>30.07	31.31 ± 22.4<br>35.33 ± 16.93	0.582
No pal<br>Pal246	2<br>2	4<br>4	0<br>0	0.22<br>0	0<br>0	0.07 ± 0.32<br>0 ± 0	nd
No pal<br>Pal246	3<br>3	1<br>1	0<br>0.17	0<br>0.67	0<br>0.36	0 ± 0<br>0.4 ± 0.61	nd
No pal<br>Pal246	3<br>3	2<br>2	0<br>0.35	0.67<br>0.67	1.09<br>1.09	0.58 ± 1.36<br>0.7 ± 0.92	0.781
No pal<br>Pal246	3<br>3	3<br>3	2.79<br>0.87	0.67<br>2.22	0.36<br>1.81	1.27 ± 3.28<br>1.63 ± 1.72	0.702
No pal<br>Pal246	4<br>4	2<br>2	0.17<br>0.17	0<br>0	0<br>0.36	0.06 ± 0.25<br>0.18 ± 0.45	0.387
No pal<br>Pal246	4<br>4	3<br>3	0<br>0	0<br>0	0.36<br>0	0.12 ± 0.52<br>0	nd
No pal<br>Pal246	4<br>4	4<br>4	0.7<br>1.4	0.44<br>0.89	0.14<br>0	0.86 ± 1.3<br>0.76 ± 1.75	0.852

Supplemental References

Blattner, F.R., Plunkett, G.3., Bloch, C.A., Perna, N.T., Burland, V., Riley, M., Collado-Vides, J., Glasner, J.D., Rode, C.K., Mayhew, G.F. et al. (1997). The complete genome sequence of *Escherichia coli* K-12. Science. *277,* 1453-1474.

Darmon, E., Lopez-Vernaza, M.A., Helness, A.C., Borking, A., Wilson, E., Thacker, Z., Wardrope, L., and Leach, D.R. (2007). SbcCD regulation and localization in *Escherichia coli*. J.Bacteriol. *189,* 6686-6694.

Khlebnikov, A., Datsenko, K.A., Skaug, T., Wanner, B.L., and Keasling, J.D. (2001). Homogeneous expression of the P(BAD) promoter in *Escherichia coli* by constitutive expression of the low-affinity high-capacity AraE transporter. Microbiology. *147,* 3241-3247.

Mahdi, A.A., Sharples, G.J., Mandal, T.N., and Lloyd, R.G. (1996). Holliday junction resolvases encoded by homologous *rusA* genes in *Escherichia coli* K-12 and phage 82. J.Mol.Biol. *257,* 561-573.

Merlin, C., McAteer, S., and Masters, M. (2002). Tools for characterization of *Escherichia coli* genes of unknown function. J.Bacteriol. *184,* 4573-4581.

Miller J.H. (1992). A short course in bacterial genetics (New York: Cold Spring Harbor Laboratory Press).

Pinder, D.J., Blake, C.E., Lindsey, J.C., and Leach, D.R. (1998). Replication strand preference for deletions associated with DNA palindromes. Mol.Microbiol. *28*, 719-727.

Wertman, K.F., Wyman, A.R., and Botstein, D. (1986). Host/vector interactions which affect the viability of recombinant phage lambda clones. Gene. *49*, 253-262.

Zahra, R., Blackwood, J.K., Sales, J., and Leach, D.R. (2007). Proofreading and secondary structure processing determine the orientation dependence of CAG x CTG trinucleotide repeat instability in *Escherichia coli*. Genetics. *176*, 27-41.
